# The effect of educational intervention based on Pender's health promotion model on quality of life and health promotion in patients with heart failure: an experimental study

**DOI:** 10.1186/s12872-021-02294-x

**Published:** 2021-10-05

**Authors:** Hossein Habibzadeh, Akram Shariati, Farshad Mohammadi, Salman Babayi

**Affiliations:** 1 School of Nursing and Midwifery, Urmia University of Medical Sciences, Urmia, Iran; 2 Department of Cardiology, School of Medicine, Urmia University of Medical Sciences, Urmia, Iran; 3 Student’s Research Committee of Nursing and Midwifery Faculty, School of Nursing and Midwifery, Urmia University of Medical Sciences, P.O Box: 575611- 5111 Urmia, Iran; 4 Department of Mathematics, Faculty of Science, Urmia University, Urmia, Iran

**Keywords:** Health promotion, Quality of life, Heart failure, Educational intervention

## Abstract

**Background:**

Heart failure is a common and chronic heart condition with high prevalence and mortality rates. This debilitating disease as an important predictor of health outcomes is directly related to patients' quality of life. Given that one of the main goals of heart failure treatment is to promote patients' quality of life and health status, conducting effective nursing interventions seems to be necessary in this regard. Therefore, the present study aimed to determine the effect of educational intervention based on Pender's health promotion model on quality of life and health promotion in patients with heart failure.

**Methods:**

This is an experimental study in which a total of 80 patients with heart failure were recruited and randomly allocated to two groups of intervention and control (*n* = 40 in each group). The educational program was designed based on Pender's health promotion model and then provided for the patients in the intervention four subgroups (10 person in each group) during six sessions. Data were collected at three time-points of before, immediately after, and three months after the intervention using a demographic questionnaire, the Minnesota Living with Heart Failure Questionnaire (MLHFQ), and the Health-Promoting Lifestyle Profile II (HPLP-II). Data were then analyzed using SPSS Statistics for Windows, version 17.0 (SPSS Inc., Chicago, Ill., USA) and *p* value less than 0.05 was taken as statistically significant.

**Results:**

Based on the results of the present study, no statistically significant difference was shown in terms of demographic characteristics between the two groups. It was also indicated that there was a statistically significant difference in the mean scores of all dimensions of quality of life (except in the physical dimension) between the two groups so that the overall mean score of quality of life increased significantly in the intervention group after the intervention (*p* < .05). Moreover, there were significant increases in the mean scores of health-promoting behaviors (except in the domain of physical activity) in the intervention group compared to the control group (*p* < .05) after intervention.

**Conclusions:**

This study demonstrates a trend that Pender's health promotion model is effective in improving the quality of life of patients with heart failure except of the physical dimension, and strengthening their health-promoting behaviors in all dimensions except of the physical activity dimension.

## Background

Heart Failure (HF) is a common and chronic cardiovascular disease with a high prevalence and mortality rate [1]. HF was previously known as a disease of the elderly, although the results of previous studies in this area show that its incidence and prevalence rates are considerably increasing among the young population. It is noteworthy that the prevalence of HF is still directly related to aging [2–4]. About 65 million people are currently suffering from HF worldwide. Moreover, about 4.2% of the general population in developed countries is affected by this disease, while its prevalence rate among people over 65 years of age is reported to be 11.8% in these countries. Meanwhile, HF patients with preserved Ejection Fraction (EF) may be easily ignored in the above statistics [1]. In Iran, the prevalence of HF is reported to be 9.4% in women and 7.9% in men [5]. Regarding poor prognosis, high mortality rate, and frequent readmissions in HF patients all of which can place a heavy burden on the healthcare systems, this disease has become a challenging health problem [5, 6].

Not only do clinical manifestations of this disease (i.e. shortness of breath, limb swelling, and decreased tolerance to activities of daily living associated with the chronic nature of the disease) negatively affect the biological, social, economic, and psychological dimensions of patients' lives [7] and drain the financial sources of healthcare systems [8], but also they can considerably lower patients' Quality of Life (QOL) [9]. In this regard, poor QOL leads to prolonged length of hospital stay, increases the mortality rate, and imposes high costs to the health system, patients, and their families. Therefore, the QOL assessment and its improvement can promote the state of health in HF patients and increase their survival rate [10]. The QOL as a multi-dimensional concept is influenced by many factors [11]. In most cases, poor QOL in HF patients stems from physical and psychological symptoms of the disease as well as social constraints in many areas such as occupation and family [12, 13]. Therefore, the researchers aimed to conduct a safe, feasible, and cost-effective educational intervention with intense concentration on the physical, psychological, and socio-economic dimensions of patients' activities.

Despite all efforts, studies in this area have indicated that HF patients do not have a standard QOL. Thus health care providers, especially nurses, should provide the necessary conditions to improve patients' QOL and health status [14–16]. It should be also mentioned that nurses constitute more than 70% of the healthcare professionals and play a major role in patient education. Nurses also have more communication with patients and their families and spend a great amount of time on patient care. Accordingly, they have a good opportunity to conduct patient education and evaluate its consequences. On the other hand, QOL is one of the concepts closely related to nursing and nurses have always striven to improve the patients' QOL through the provision of health care and participation in nursing research projects [17]. Despite that medical advances have improved patients' health outcomes, nurses still have to play their particular roles alongside [8].

Nurses have of course paid attention to this issue and also presented different models in this area with regard to this point that one of the most effective ways to improve QOL in HF patients is the use of model-based educational intervention [18, 19]. In this regard, one of the most comprehensive and widely used models of patient education provided by nurses is Pender's health promotion model [20]. This model was developed by Nola J. Pender in 1982 with an emphasis on health promotion and empowering individuals to achieve a good state of health. This model is a theoretical perspective that determines the health factors and their relationships with health-promoting behaviors so that it can lead to considerable improvement in patients' QOL and health status [18]. In addition to having six domains of health promotion including nutrition, physical activity, health responsibility, stress management, interpersonal relations, and spiritual growth, this model measures the factors affecting the above dimensions. These factors consist of the perceived advantages and barriers to health behavior, perceived self-efficacy, and influential interpersonal resources [21]. Perceived advantages of health behavior and self-efficacy can increase commitment to the behavior and reduce perceived barriers to it. In this regard, family, friends, and healthcare providers are known as influential interpersonal resources that can increase or decrease commitment to the behavior [22]. Despite that the effectiveness of Pender's health promotion model in education has been confirmed in many studies [23, 24], the application of this model as a comprehensive, regular, and cost-effective method of patient education has still received less attention. Providing education to promote patients' quality of life and health status is an important step in raising awareness and involving patients, which is strongly influenced by socio-economic and lifestyle determinants. To achieve this goal and influence the consequences of the disease, the Pender's health promotion model can be used. We chose Pender’s model because of our background in heart failure, we have the same perception to promote patients' quality of life and health status as she did. Therefore, we tried to communicate with this model and by considering the effective and underlying factors, draw a way for more participation of these patients in self-care and making better decisions. Pender states that patients' quality of life could be improved by preventing acute or chronic health problems [25]. She describes the purpose of this model as assisting nurses in understanding the main determinants of health behaviors as a basis for behavioral counseling to promote a healthy lifestyle [26].

Based on the literature review, in the majority of studies on patient education, the researchers used non-nursing models to perform the interventions. However, many studies investigated the effect of educational interventions on QOL in patients with HF. Furthermore, most of the recent studies in this area have been conducted in developed countries. Therefore, it seems that conducting a study in developing countries can be a fundamental step to strengthen the evidence in this area and through this way, more generalizable results can be obtained about the effect of educational intervention based on this model on the domains of health promotion and patients' QOL. Besides, poor QOL in Iranian patients with HF [27] and poor patient compliance indicate the lack of systematic, integrated, and planned patient education. Concerning the lack of outpatient care facilities in Iran [28], the need for an affordable, safe, and planned method of patient education is increasingly felt in medical centers. Therefore, regarding the increasing number of HF patients resulting from aging and population growth [1] as well as the importance of using nurse-led patient education models as a framework for the design of patient education programs, this study was conducted to determine the effect of educational intervention based on Pender's health promotion model on QOL and health promotion in HF patients.

## Methods

### Study design

This is an experimental study with a pretest–posttest control group design and a three-month follow-up.

### Study setting and participants

In this study, a total of 80 HF patients referred to the clinic of Seyyed-Al-Shohada Heart Center affiliated to Urmia University of Medical Sciences were recruited using systematic random sampling. Concerning that this medical center is the only cardiology hospital in the northwest of Iran, the majority of patients with cardiac diseases are referred to this center. So the researchers selected this center as the study setting in order to have more and easy access to the target population of the study.

Inclusion criteria consisted of the followings: (a) having a medical record in the clinic of the heart center, (b) definitive diagnosis of HF (Classes I and II) by a cardiologist, (c) having at least a three-month history of HF, (d) having residency in Urmia (concerning easy access to the patient), (e) willingness to participate in the study, and (f) the ability to attend all training sessions. In contrast, exclusion criteria consisted of the followings: (a) unwillingness to continue participation in the study, (b) lack of access to the patient, (c) having a mental health disorder, (d) being physically dependent on others, and (e) being absent from more than two training sessions.

### Sample Size

Considering the confidence interval of 95% and the test's power of 84%, the minimum sample size was considered to be 76 (n = 38 in each group). To calculate the minimum sample size, the formula $n \ge \frac{{2\sigma^{*2} \left( {z_{\alpha /2} + z_{\beta } } \right)^{2} }}{{\Delta^{2} }}$ was utilized in accordance with the below values [29].

- Probability of making a type I error (α): $\alpha = 0.05 \Rightarrow z_{\alpha } = 1.96$
- Test's power: $$1 - \beta = 0.80 \Rightarrow z_{\beta } = 0.84$$
- The difference in clinical significance: ${\upmu }_{1} - {\upmu }_{2} = 7.88$
- $${\text{Effect size}} = |\Delta|/\sigma^{*}=7.88/20.51=0.8$$  

Regarding the probability of 15% sample attrition, the final sample size was considered to be 88 (n = 44 in each group). After preparing a list of eligible patients and tossing a coin, those who received treatment in the morning and the evening shift were randomly allocated to the intervention and the control group, respectively. The total number of patients in the morning and the evening shift was separately extracted and recorded in a list. The sampling interval (K) was determined using the following formula and the sampling begun by choosing a number from the list at random and then every Kth number in the frame was selected.

$$K = \frac{{N \left( {\text{population size}} \right){ }}}{{n \left( {\text{sample size}} \right)}}$$

$$K \left( {morning\,shift} \right) = \frac{{1100{ }}}{44} = 25 \quad K \left( {evening\,shift} \right) = \frac{{875{ }}}{44} = 20$$

Finally, a number of 44 patients in the morning shift were assigned to the intervention group and a number of 44 patients in the evening shift were assigned to the control group. It should be also noted that patients in both groups were not informed of their allocation to the intervention or the control group. Regarding the loss of 8 samples during the study (resulted from patient relocation, lack of access to the patient, incomplete questionnaire, and the absence from more than two training sessions), the final sample size decreased to 80 for both groups (*n* = 40 in each group).

### Intervention

In order to provide comfort for the participants and prevent disruption to the completion of the questionnaires, the pretest was conducted in a room with a comfortable and quiet atmosphere located in the clinic of the heart center. The second author of the study helped the participants who were illiterate to fill out the questionnaires. Then, the educational content was prepared based on valid sources and Pender's health promotion model. This content was also formed in accordance with the study objectives and strategies to improve the domains of Pender's model. Then the prepared content was presented to the patients in the intervention group, while patients in the control group received no training. In order to achieve better effect of the intervention, improve patients' participation, prevent irregularity, and run better management of the training sessions, the intervention group was divided into four sub-groups (n = 10 in each sub-group) and the training was provided using lecture and group discussion in the conference room of the heart center. Each sub-group was provided with six one-hour sessions. The question and answer method was utilized to have a better understanding of the educational content and prevent one-way teaching. In this study, materials such as whiteboard, PowerPoint slides, and projector were used to make the training more effective. In order to prevent data contamination between the two groups, all training sessions were held in the morning. Moreover, to comply with ethical principles, the educational content was provided for the control group in the form of an educational booklet at the end of the study. The questionnaires were re-completed immediately and three months after the intervention by the patients in both groups and data were then analyzed.

### Data collection

In this study, data collection was conducted using a demographic questionnaire, the Minnesota Living with Heart Failure Questionnaire (MLHFQ), and the Health-Promoting Lifestyle Profile II (HPLP-II).

The MLHFQ is a self-administered tool developed by Thomas S. Rector (1984) for measuring the QOL in HF patients. This questionnaire is the most widely used tool for assessing the level of QOL in patients with HF and consists of 21 items in the physical (12 items), emotional/psychological (5 items), and socio-economic (4 items) dimensions. Each item is scored on a 6-point Likert scale, so that the overall score ranges from 0 to 105 and the greater score indicates a higher level of QOL. The validity and reliability of this questionnaire were reported to be high in all studies conducted in this area. The Persian version of this questionnaire has examined by Eskandari et al. [30] as they assessed its reliability using internal consistency and reported Cronbach's alpha of *α* = 0.95 for the whole questionnaire. They also confirmed the validity of the tool.

The HPLP-II is an applicable tool for measuring and assessing health-promoting behaviors. This questionnaire has been developed by Walker et al. [31] based on Pender's health promotion model and consists of 52 items in six domains of health responsibility (13 items), nutrition (8 items), physical activity (8 items), stress management (5 items), interpersonal relations (8 items), and spiritual growth (10 items). This questionnaire is scored on a 4-point Likert scale from "Never = 1" to "Routinely = 4", so that the overall score ranges from 52 to 208. Walker and Hill [32] assessed the reliability of this tool as they reported the Cronbach's alpha of *α* = 0.94 for the whole questionnaire. Mohammadi Zeidi et al. [33] evaluated the validity and reliability of the Persian version of this questionnaire so that they reported the Cronbach's alpha for the whole questionnaire to be *α* = 0.82 and confirmed its validity as well.

### Data analysis

Data were first entered into SPSS Statistics for Windows, version 17.0 (SPSS Inc., Chicago, Ill., USA) and then analyzed using descriptive (mean, standard deviation, percentage, and frequency) and inferential statistics (independent-samples t-test, chi-squared test, and Repeated measures ANOVA).

### Educational content

The educational content presented in this study was based on the domains of Pender's health promotion model including nutrition, physical activity, health responsibility, stress management, interpersonal relations, and spiritual growth. This model also plays an essential role in improving the QOL [34]. In this model, emotions about health behavior such as perceived advantages and barriers, perceived self-efficacy, and influential interpersonal resources directly affect behavior. In this study, patient education was conducted with intense concentration on the above factors. For instance, in the domain of physical activity, the patients were provided with explanations on the advantages of health behavior (e.g. physical activity may reduce the number of hospitalizations), perceived barriers (e.g. cost-cutting strategies), perceived self-efficacy (e.g. using one's own abilities), and influential interpersonal resources (e.g. using the help of family and friends in doing physical activity). These factors were considered in all domains and the patients were also asked to follow the behavior modification program according to the items announced at the end of each session. This content was assessed and approved quantitatively and qualitatively by four faculty members (two nursing faculty and two cardiologists) (Table 1).

**Table 1:** Content of the educational intervention

Session no.	Educational content based on dimensions	Goals based on model constructs	Teaching materials	Teaching method
1st	Introducing patients and educator, assessment of patients' needs, and familiarizing the patients with their health condition (definition of the disease, causes, signs, symptoms, and complications)	(a) Investigate previous related behavior and the causes of previous success (b) Increase perceived benefits (c) Reduce perceived barriers	Whiteboard, board marker, computer, projector, PowerPoint slides	Lecture, group discussion, and question and answer
2nd	Reviewing the content of the previous session, enumerating modifiable and non-modifiable risk factors for HF, and giving a presentation on healthy and unhealthy behaviors affecting the heart health	(d) Increase perception of self-efficacy (e) Increase understanding of social support	Whiteboard, board marker, computer, projector, PowerPoint slides	Lecture, group discussion, and question and answer
3rd	Reviewing, the role of regular physical activity and nutrition	(f) Improve behavior-related feelings	Whiteboard, board marker, computer, projector, PowerPoint slides	Lecture, group discussion, and question and answer
4th	Reviewing, the role of interpersonal relations and stress management	(g) Analyzes the situation and living environment	Whiteboard, board marker, computer, projector, PowerPoint slides	Lecture, group discussion, and question and answer
5th	Reviewing, the role of health responsibility and spiritual growth	(h) Commitment to the action plan and its maintenance	Whiteboard, board marker, computer, projector, PowerPoint slides	Lecture, group discussion, and question and answer
6th	Reviewing and summarizing the content of previous sessions and answering patients' questions	(i) Raise awareness of urgent competitive preferences and strategies to deal with them	Whiteboard, board marker, computer, projector, PowerPoint slides	Lecture, group discussion, and question and answer

## Results

### Demographic characteristics

A total of 80 patients with HF participated in the present study and were randomly divided into two groups of intervention and control (*n* = 40 in each group). The mean age of the participants in the intervention and the control group was 56.8 ± 11.11 and 57.9 ± 9.75, respectively. The majority of the participants in the intervention (60%) and the control group (65%) were male and the rest were female. Based on the results, there was no statistically significant difference in terms of demographic characteristics between the two groups (*p* > 0.05). In other words, the two groups were homogenous in terms of demographic characteristics (Table 2).

**Table 2:** Demographic and clinical characteristics between the two groups (n = 80)

Characteristics	Intervention group	Control group	*p* value
Age, mean (SD)	56.8 (11.11)	57.9 (9.57)	0.9^a^
Gender (n, %)			
Male	14 (35%)	15 (37.5%)	0.295^b^
Female	26 (65%)	25 (62.5%)	
Educational level (n, %)			
Less than diploma	26 (65%)	25 (62.5%)	0.386^b^
Diploma	8 (20%)	10 (25%)	
Higher education	6 (15%)	5 (12.5%)	
Job (n, %)			
Employed	26 (65%)	26 (65%)	0.688^b^
Unemployed	5 (12.5%)	4 (10%)	
Retired	9 (22.5%)	10 (25%)	
Marital status (n, %)			
Single and widow	2 (5%)	8 (20%)	0.089^b^
Married	38 (95%)	32 (80%)	
Smoking (n, %)			
Yes	11 (27.5%)	10 (25%)	0.645^b^
No	29 (72.5%)	30 (75%)	
Previous hospitalization (n, %)			
Yes	24 (60%)	21 (53%)	0.476^b^
No	16 (40%)	19 (47%)	

^a^Independent samples t-test

^b^Chi-square

### QOL

**Fig. 1:**
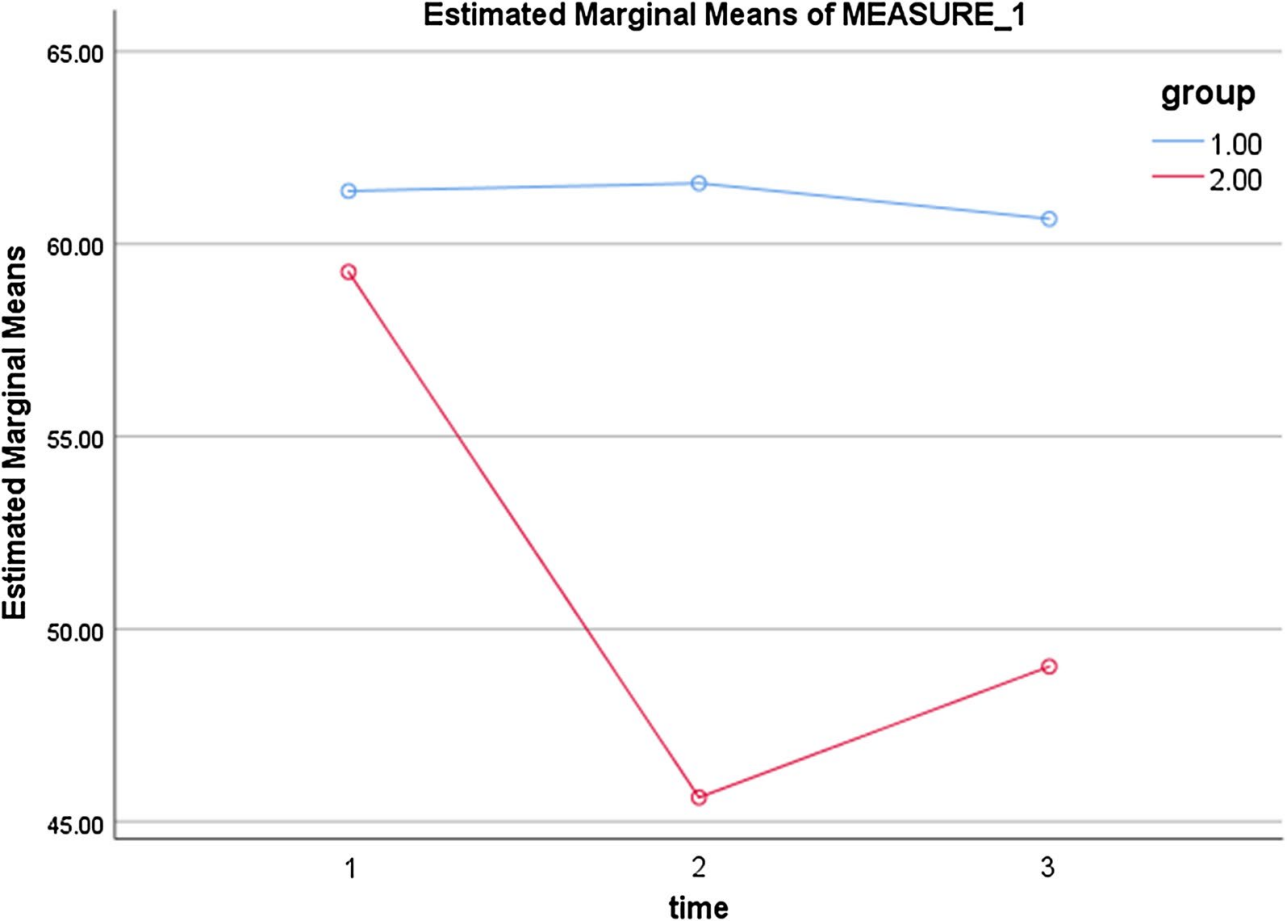
Changes in the mean score of QOL at the three time points between the intervention and control groups (group 1.00: control, group 2.00: intervention, 1: before, 2: immediately after, and 3: three months after the intervention)

The results of the independent-samples t-test showed that there was no statistically significant difference between the two groups in the mean score of QOL before the intervention. However, the difference in the mean scores of overall QOL and its psychological and socio-economic dimensions was found to be statistically significant between the two groups at two time points of immediately and three months after the intervention (*p* < 0.05). In other words, the mean score of QOL decreased significantly in the intervention group compared to the control group after the intervention. Moreover, the mean score of QOL at the time point of the three months after the intervention was higher than that at the time point of immediately after the intervention (Table 3). Also, based on the results, the effect of time and the interaction of time and group (Table 4), and the difference between the two groups, were statistically significant (Table 5). Figure 1 shows the changes of both groups over time.

**Table 3:** Comparison of quality of life scores between intervention and control groups

Quality of life dimensions	Before the intervention	Immediately after the intervention	3 month after the intervention	*p* value
	Mean (SD)	Mean (SD)	Mean (SD)	
Physical				
In. G	25.65 (4.98)	20.47 (3.30)	21.82 (3.57)	^**^p = 0.127
Co. G	26.35 (4.22)	25.25 (4.95)	25.05 (5.22)	^**^p = 0.433
*p* value^*^	*p* = 0.44	*p* = 0.076	*p* = 0.53	
Emotional/psychological				
In. G	18.60 (4.22)	14.70 (2.57)	14.70 (1.77)	^**^p = 0.000
Co. G	18.87 (3.68)	19.85 (3.80)	19.40 (3.39)	^**^p = 0.448
*p* value^*^	*p* = 0.662	*p* = 0.021	*p* = 0.001	
Socio-economical				
In. G	15.02 (3.88)	10.45 (1.88)	12.5 (1.64)	^**^p = 0.000
Co. G	16.15 (3.57)	16.47 (3.61)	16.20 (2.80)	^**^p = 0.897
*p* value^*^	*p* = 0.847	*p* = 0.001	*p* = 0.002	
Total				
In. G	59.27 (7.51)	45.62 (4.27)	49.02 (4.20)	^**^p = 0.001
Co. G	61.37 (6.26)	61.57 (6.86)	60.65 (6.81)	^**^p = 0.808
*p* value^*^	*p* = 0.180	*p* = 0.004	*p* = 0.021	

In. G, intervention group; Co. G: control group

^*^The independent samples t-test was used

^**^The repeated measures ANOVA test was used

**Table 4:** Multivariate tests of QOL (Time, time * group interaction)

Effect	Value	F	Hypothesis df	Error df	Sig	Partial eta squared
Time						
Pillai's trace	.384	24.024	2.000	77.000	.000	.384
Wilks' lambda	.616	24.024	2.000	77.000	.000	.384
Hotelling's trace	.624	24.024	2.000	77.000	.000	.384
Roy's largest root	.624	24.024	2.000	77.000	.000	.384
time * group						
Pillai's trace	.372	22.854	2.000	77.000	.000	.372
Wilks' lambda	.628	22.854	2.000	77.000	.000	.372
Hotelling's trace	.594	22.854	2.000	77.000	.000	.372
Roy's largest root	.594	22.854	2.000	77.000	.000	.372

**Table 5:** Tests of between-subjects effects (group)

Source	Type III sum of squares	Df	Mean square	F	Sig	Partial eta squared
Intercept	759,487.504	1	759,487.504	23,663.276	.000	.997
Group	5870.704	1	5870.704	182.913	.000	.701
Error	2503.458	78	32.096

### Health-promoting behaviors

**Fig. 2:**
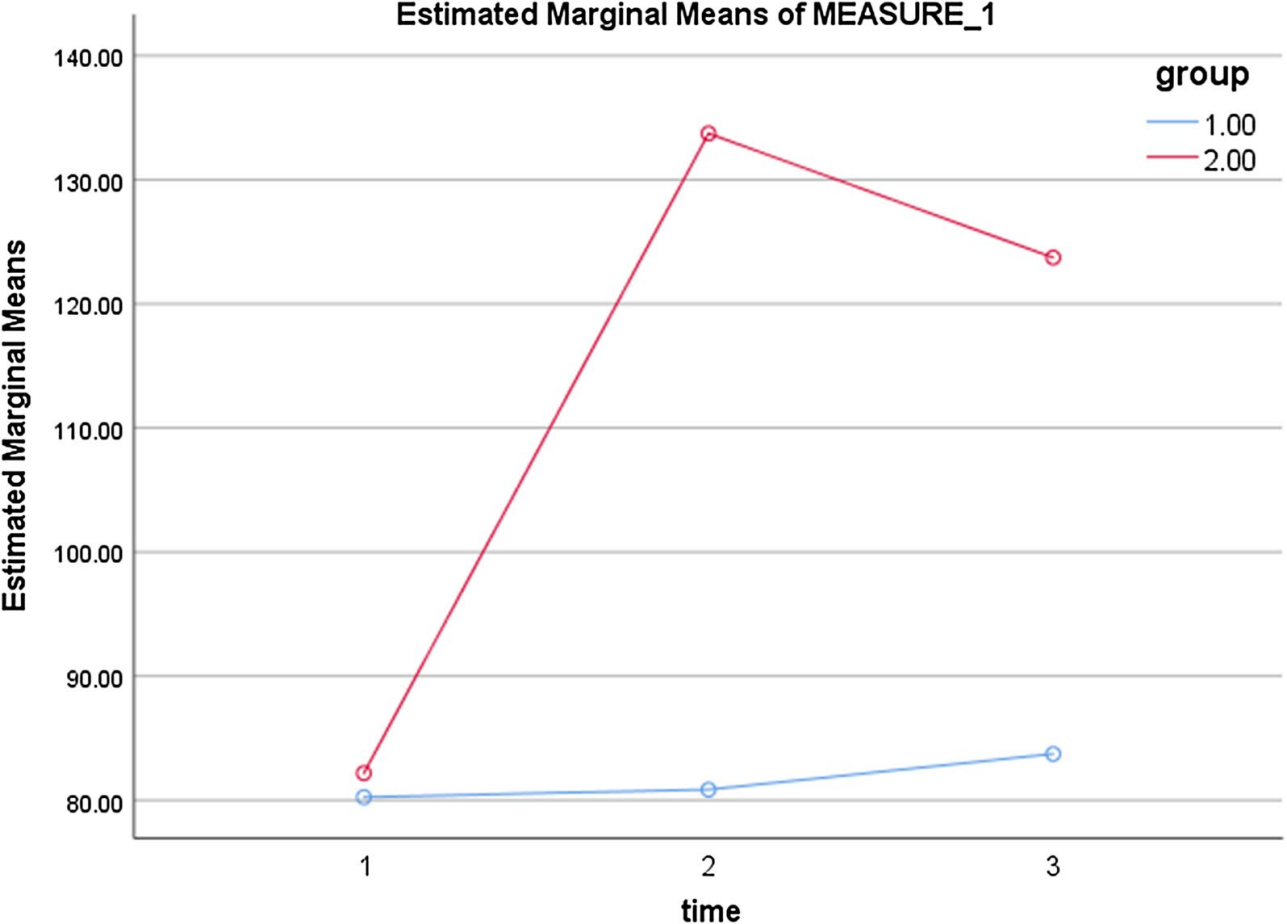
Changes in the mean score of HPLP-II Dimensions at three time points between intervention and control groups (group 1.00: control, group 2.00: intervention, 1: before, 2: immediately after, and 3: three months after the intervention)

The results of the independent-samples t-test indicated no statistically significant difference in the mean scores of the domains of nutrition, health responsibility, stress management, interpersonal relations, and spiritual growth between the two groups before the intervention. However, after the intervention, the mean scores of the above domains increased significantly in the intervention group (*p* < 0.05). The mean score of the domain of physical activity also increased in the intervention group but this increase was not shown to be statistically significant (*p* > 0.05) (Table 6). Also, the effect of time and the interaction of time and group (Table 7), and the difference between the two groups, were statistically significant (Table 8). Figure 2 shows the changes of both groups over time.

**Table 6:** Comparison of HPLP-II dimensions scores between intervention and control groups

HPLP-II dimensions	Before the intervention	Immediately after the intervention	3 month after the intervention	*p* value
	Mean (SD)	Mean (SD)	Mean (SD)	
Nutrition				
In. G	12.35 (3.59)	23.22 (5.13)	18.52 (3.94)	^**^p = 0.000
Co. G	11.85 (2.66)	10.97 (3.33)	12.07 (2.56)	^**^p = 0.20
*p* value^*^	*p* = 0.175	*p* = 0.022	*p* = 0.008	
Physical activity				
In. G	9.90 (2.87)	9.70 (2.40)	10.01 (2.49)	^**^p = 0.871
Co. G	10.27 (2.27)	9.27 (2.25)	9.80 (2.05)	^**^p = 0.130
*p* value^*^	*p* = 0.685	*p* = 0.256	*p* = 0.357	
Health responsibility				
In. G	17.95 (3.24)	34.17 (5.48)	33.15 (6.02)	^**^p = 0.001
Co. G	17.12 (2.94)	17.30 (3.25)	16.72 (2.84)	^**^p = 0.684
*p* value^*^	*p* = 0.588	*p* = 0.007	*p* = 0.001	
Stress management				
In. G	10.90 (3.02)	14.62 (4.38)	10.42 (2.80)	^**^p = 0.001
Co. G	10.01 (2.40)	9.55 (2.62)	10.65 (2.88)	^**^p = 0.179
*p* value^*^	*p* = 0.280	*p* = 0.011	*p* = 0.849	
Interpersonal relations				
In. G	10.80 (2.46)	21.52 (2.10)	19.20 (2.02)	^**^p = 0.000
Co. G	10.70 (2.94)	14.20 (4.86)	14.30 (5.34)	^**^p = 0.000
*p* value^*^	*p* = 0.197	*p* = 0.000	*p* = 0.000	
Spiritual growth				
In. G	20.27 (4.07)	30.47 (5.64)	32.42 (5.33)	^**^p = 0.000
Co. G	20.30 (3.01)	19.55 (2.95)	20.17 (2.89)	^**^p = 0.479
*p* value^*^	*p* = 0.092	*p* = 0.006	*p* = 0.001	
Total				
In. G	82.17 (8.87)	133.72 (10.68)	123.72 (10.57)	^**^p = 0.000
Co. G	80.25 (6.39)	80.85 (6.66)	83.72 (6.61)	^**^p = 0.404
*p* value^*^	*p* = 0.244	*p* = 0.011	*p* = 0.017	

In. G, intervention group; Co. G, control group

^*^The independent samples t-test was used

^**^The Repeated measures ANOVA test was used

**Table 7:** Multivariate tests of HPLP-II dimensions (Time, time * group interaction)

Effect	Value	F	Hypothesis df	Error df	Sig	Partial eta squared
Time						
Pillai's trace	.871	259.174	2.000	77.000	.000	.871
Wilks' lambda	.129	259.174	2.000	77.000	.000	.871
Hotelling's trace	6.732	259.174	2.000	77.000	.000	.871
Roy's largest root	6.732	259.174	2.000	77.000	.000	.871
time * group						
Pillai's trace	.854	225.708	2.000	77.000	.000	.854
Wilks' lambda	.146	225.708	2.000	77.000	.000	.854
Hotelling's trace	5.863	225.708	2.000	77.000	.000	.854
Roy's largest root	5.863	225.708	2.000	77.000	.000	.854

**Table 8:** Tests of between-subjects effects (group)

Source	Type III sum of squares	df	Mean square	F	Sig	Partial eta squared
Intercept	2,277,212.017	1	2,277,212.017	32,308.420	.000	.998
Group	59,913.600	1	59,913.600	850.037	.000	.916
Error	5497.717	78	70.484

## Discussion

Accordingly, the present study aimed to determine the effect of educational intervention based on Pender's health promotion model on QOL and health promotion in patients with HF. Even though this study had few limitations, the educational intervention based on Pender's health-promotion model improved QOL in patients with HF. Based upon the results; it was clearly found that the level of QOL significantly increased after the educational intervention in the intervention group compared to the control group. The effect of educational interventions on QOL in HF patients has been also assessed in other studies. In this regard, Osaba et al. [35] has confirmed the positive effect of educational intervention on QOL. Smeulders et al. [36] came to this result that nursing educational interventions have a positive effect on QOL in patients with HF, which is consistent with the results of our study. However, they assessed the effect of educational programs on the QOL in a small population, while we addressed this limitation in our study.

Based on the results, the difference in the mean score of physical dimension was not found to be statistically significant between the two groups after the intervention. It seems that making a change in terms of this dimension is a bit difficult for HF patients and requires more interventions. Regarding the causes, it can be stated that respiratory complications caused by HF can limit the level of physical activity, threaten the patient's independence, and cause him/her to be dependent on others in this regard [37]. Karataş and Polat [20] found that factors such as the lack of social support, fear of heart attack, risk of injury and falls, lack of a previous habit of doing physical activity, lack of information, and functional limitations constitute barriers to physical activity in patients with coronary artery disease. von Haehling et al. [38] showed that their interventions had no considerable impact on the improvement of patients' physical performance so that they recommended using other strategies to improve it. The results also indicated that the mean score of the psychological dimension of QOL in the intervention group was higher than in the control group after the educational intervention. In line with the results of our study, Baghaei et al. [39] demonstrated that the mean score of the socio-economic dimension of QOL increased significantly after the educational intervention in the intervention group compared to the control group. The results of the study by Shumaker et al. [40] on the relationship between social support and QOL in HF patients are showed to be consistent with the results of our study.

Based on the findings of our study, it was revealed that the level of QOL increased in HF patients, but it should be mentioned that the mean score of QOL was not within a normal range in both groups and previous studies have reported similar results [41–43]. Therefore, nurses have a key role in improving the level of QOL in these patients. Moreover, one of the main goals of HF treatment is to maximize the patients' QOL, so nurses as important members of the healthcare team should not spare any effort to safely do so. Of course, it is abundantly clear that there are many influential factors in assessing patients' QOL, including study setting, the mean age of participants, gender, HF classification, level of patients' EF, and socio-economic status. Despite the differences in the above factors, the fact that many studies in this area have not considered the level of QOL to be standard enough can persuade nurses to conduct further studies in this regard.

The results also showed a significant increase in the mean scores of all domains of health-promoting behaviors except in physical activity (*p* < 0.05). The mean score of physical activity increased, but this increase was not found to be statistically significant (*p* > 0.05). Consequently, it was concluded that the educational intervention caused no effect on both the physical dimension of QOL and the domain of physical activity of health-promoting behaviors. The above result is in line with the results of the studies by Rahimian et al. [44] and Srisoongnern et al. [45]. Given the low mean scores of the emotional/psychological dimension of QOL among participants, it can be concluded that their visions and viewpoints were impaired and they lacked the necessary motivation and positive feeling to do physical activity. Furthermore, according to patients' feedback during the study, it seems that the negative belief and attitude towards physical activity have been formed and even stabilized in HF patients as it would not improve using short-term education and requires long-term interventions to be addressed. Therefore, the lack of a statistically significant difference in the mean score of the physical dimension of QOL is expected since it can be affected by cultural issues, gender, individual habits, and other factors [16]. In line with the results of our study, Mohammadipour et al. [46] showed no significant difference in the mean score of physical activity between the two groups after the educational intervention. Regarding the fact that patients who are less physically active have a poor prognosis [4], other researchers are recommended to conduct long-term studies and utilize an interdisciplinary team for encouraging patients to do physical activity, understand the advantages of this behavior, and surmount the obstacles in this way.

Based on the results of our study, it was revealed that there was a statistically significant difference in the mean score of nutrition between the two groups after the intervention. It was also indicated that the mean scores of health responsibility increased significantly in the intervention group compared to the control group after the intervention. The above results are consistent with the results of the studies by Omidi et al. [24], Mohammadipour et al. [46], and Farsi et al. [47]. In terms of stress management, the results showed a significant increase in the mean score of this dimension in the intervention group compared to the control group immediately after the intervention, although this difference was not found to be significant between the two groups at the time point of three months after the intervention. The reason for the instability of the effect of education on stress management in the intervention group can be sought in patients' lifestyles and factors affecting it, such as socio-economic status. In line with the results of the study by Omidi et al. [24], we found that the mean score of the interpersonal relations increased significantly in the intervention group compared to the control group. However, this result is inconsistent with the results of the study of Mohammadipour et al. [46]. The differences in the target population and patients' lifestyles constitute the reason behind this discrepancy in the results. In line with the results of the study by Omidi et al. [24], the difference in the mean score of spiritual growth was found to be statistically significant between the two groups after the intervention. However, this result was inconsistent with the results of the study by Mohammadipour et al. [46] since they found that there was no significant difference between the two groups in terms of spiritual growth. In this regard, it should be noted that the target population of their study was made up of diabetes patients.

In the present study, the overall mean score of health-promoting behaviors was also measured and found to be statistically significant between the two groups after the intervention. In other words, the overall mean score of health-promoting behaviors in the intervention group was higher than in the control group. Accordingly, the educational intervention based on Pender's health promotion model was approved to have a positive effect on the domains of health-promoting behaviors. In a systematic review, Mohebi et al. [48] achieved this result that Pender's health promotion model is an effective method for patient education and instructional interventions, which is consistent with the results of our study. Carreno et al. [49] also revealed that the educational intervention based on Pender's model can lead to the improvement of health behaviors in the intervention group in all domains of health promotion.

### Study limitations

Despite the positive effect of educational intervention on the dimensions of QOL and domains of health promotion, this study had some limitations. One of the limitations of this study was the mental state of the patients with regard to the chronic nature of the disease, which could affect their level of QOL and the results of the study as well. This limitation was beyond the researcher's control. In addition, concerning the fact that the study setting was a hospital, hospitalized patients might not be a good representative of the target population, since HF patients also receive health services in other health centers such as doctors' offices and clinics. Regarding the COVID-19 pandemic and related social distancing, the researcher had no access to HF patients in other centers. Moreover, given many reasons, patients with HF have frequent hospital admissions, which can affect their willingness to participate in researches. Another limitation was the lack of measurement of long-term clinical effects of the educational intervention such as mortality and hospitalization rates, treatment costs, patient satisfaction, and patients' social performance. This limitation can be also overcome by following up patients' status and conducting another study in the future.

## Conclusion

The results of the present study concealed that educational interventions conducted by nurses based on Pender's health promotion model were effective in improving the psychological and socio-economic dimensions of QOL in patients with HF. However, these interventions were not shown to be effective in modifying patients' behavior in the physical dimension of QOL and this required more interventions. Moreover, the level of health-promoting behaviors was significantly improved in the domains of nutrition, health responsibility, stress management, interpersonal relations, and spiritual growth, which in turn led to a significant increase in patients' QOL. However, no significant increase was observed in the level of physical activity and the explanations provided in training sessions on the perceived advantages and barriers to health behavior did not persuade patients to modify their behavior in this area. The maximum improvement in the QOL can be considerably effective in reducing hospital readmissions and mortality rates, lightening the financial burden of health care, increasing health professionals' job satisfaction, and reducing their workload. Considering the above, nurses can design and implement theoretical and practical education programs using the health promotion model and ultimately take effective steps in promoting HF patients' health status and improving their level of QOL. The educational interventions do not endanger patients' safety and can be implemented with the least facilities. Besides, nurses and health workers are always able to perform these interventions. Considering the above, it is recommended to conduct more in-depth studies on different target populations in other geographical areas and medical centers. If the results of future studies confirm the positive effect of this type of education, this method can be utilized as a systematic, planned, and codified educational approach in health centers to promote the level of QOL in HF patients.

### Practice implications

Nurses and public health manager can utilize this model to develop and implement educational programs in clinical settings and take effective steps in promoting health status and improving the QoL in HF patients. Furthermore, maximizing the QoL in these patients can be effective in reducing hospital readmission, lightening the financial burden of health care, increasing nurses' job satisfaction and reducing their workload.

## Data Availability

The datasets used and/or analyzed during the current study are available from the corresponding author on reasonable request.

## Abbreviations

HF: Heart Failure
QOL: Quality of Life
HPLP-II: Health-Promoting Lifestyle Profile II
MLHFQ: Minnesota Living with Heart Failure Questionnaire
